# Oral–maxillofacial and cervical schwannomas: a retrospective cohort analysis with a rare intraosseous case

**DOI:** 10.1186/s12903-026-08073-4

**Published:** 2026-03-09

**Authors:** Xiaomin Hu, Dongxiang Wang, Zhenglong Tang

**Affiliations:** Department of Oral and Maxillofacial Surgery, Affiliated Stemmatological Hospital of Guizhou Medical University, Guiyang, 550004 China

**Keywords:** Schwannoma, Clinicopathological features, Immunohistochemistry

## Abstract

**Objective:**

To evaluate the clinical, pathological, and immunohistochemical characteristics of schwannomas (SCHs) in the oral, maxillofacial, and cervical regions to provide a reference for their diagnosis and clinical management.

**Materials and methods:**

We retrospectively analyzed 20 patients with oral, maxillofacial, and cervical SCHs treated at our institution between January 2019 and December 2025. Clinical history, presenting symptoms, and relevant auxiliary examinations were collected. Histopathological diagnoses were established using hematoxylin and eosin (H&E) staining and immunohistochemical analysis. Demographic data, clinical manifestations, surgical approaches, and pathological findings were descriptively analyzed.

**Results:**

The tumors originated predominantly from branches of the trigeminal, the lingual, and facial nerves, as well as the cervical plexus. The most common clinical presentation was a painless mass in the oral-maxillofacial region. Some patients presented with tongue sensory disturbances, masticatory dysfunction, or facial muscle weakness, depending on the affected nerve. Histopathological examination revealed typical Antoni A and Antoni B patterns with Verocay bodies, and immunohistochemical findings confirmed the diagnosis of SCH. Surgical approaches were individualized according to tumor location and size. All patients had favorable postoperative outcomes.

**Conclusions:**

SCHs of the oral and maxillofacial region and neck exhibit diverse clinical manifestations, definitive diagnosis relies on histopathological and immunohistochemical evaluation. Awareness of the tumor’s anatomical origin and associated clinical features facilitates optimal surgical planning and informed clinical decision-making.

**Supplementary Information:**

The online version contains supplementary material available at 10.1186/s12903-026-08073-4.

## Introduction

Schwannomas (SCHs) are benign tumors originating from Schwann cells and can occur in multiple anatomical sites throughout the body. Approximately 25%–45% of SCHs arise in the head and neck region, making it one of the most common locations for these tumors [1, 2]. Within the head and neck, the neck site is the most frequent presentation, whereas only about 1%–12% of cases occur within the oral cavity. The tongue is the most common intraoral location [3, 4]. Clinically, SCHs typically present as slow-growing, painless masses, which can be easily confused with other soft tissue tumors, salivary gland lesions, or odontogenic diseases, thereby complicating the diagnostic process. Moreover, systematic studies investigating the clinical and pathological differences of SCHs across various oral, maxillofacial, and cervical sites remain limited. The present study aims to analyze the clinical, pathological, and immunohistochemical features of SCHs in multiple sites of the oral cavity, maxillofacial region, and neck. In addition, we report a rare case of mandibular intraosseous SCH, providing insights for clinical diagnosis and management.

## Materials and methods

### Patient selection and inclusion criteria

This study included retrospective data from patients diagnosed with SCHs of the oral cavity, maxillofacial region, an neck at the Department of Pathology, Affiliated Stomatology Hospital of Guizhou Medical University, between January 2019 and December 2025. All the cases were identified through the pathology department registry and confirmed based on medical records and histopathological evaluation. The specimens were obtained from biopsies or surgical resections performed in the Department of Oral and Maxillofacial Surgery and the Department of Oral Mucosa, and were formalin-fixed and paraffin-embedded (FFPE).

Inclusion criteria were:

1. Tumor located in the head and neck region, including the oral cavity, maxillofacial area, or neck;
2. Histopathologically confirmed diagnosis of SCH;
3. FFPE tissue adequate for immunohistochemical analysis;
4. Complete clinical data, including preoperative symptoms, surgical records, and follow-up information.

Exclusion criteria were:

1. Tumor located outside the head and neck region;
2. Incomplete pathological data or tissue samples unsuitable for immunohistochemical analysis;
3. Missing clinical or follow-up information.

### Ethical considerations

This study was conducted in accordance with the ethical principles of the Declaration of Helsinki and was approved by the Ethics Committee of the Affiliated Stomatology Hospital of Guizhou Medical University (Approval No. 2025-Lun-Shen-46).

### Laboratory methods

The collected tissue specimens were processed using standard histological procedures. The FFPE sections were prepared and stained with hematoxylin and eosin (H&E) for morphological evaluation. Immunohistochemistry (IHC) was performed to assist in the pathological diagnosis. Eleven cases underwent IHC staining using the EnVision two-step method. The antibodies used included S100, SOX10, Calretinin, CD56, neuron-specific enolase (NSE), glial fibrillary acidic protein (GFAP), Vimentin, CD34, smooth muscle actin (SMA), Desmin, CD68, Ki-67, and additional relevant markers (AE1/AE3, Actin, Mammaglobin, Myo-D1, Calponin, CD10, STAT6, Bcl-2, β-catenin). All reagents were obtained from Dako (Agilent Technologies, Santa Clara, CA, USA), and all procedures were performed strictly according to the manufacturer’s instructions.

## Results

### Demographic and clinical characteristics

A total of 20 patients with SCHs of the oral cavity, maxillofacial region, and neck were included in this study. The patients’ ages ranged from 11 to 66 years, with a mean age of 38.5 years; 13 patients (65%) were female and seven (35%) were male. The most common tumor site was the tongue (six cases, 30%), followed by the parotid gland, neck, and lips (three cases each, 15%). The other sites included the floor of the mouth, palate, retromolar pad, chin, and mandible (one case each, 5%).

Preoperative clinical manifestations were predominantly painless, slow-growing masses (10 cases, 50%), followed by a sensation of a foreign body (seven cases, 35%), with a minority of patients reporting mild tenderness or pain (three cases, 15%). Neurological involvement was observed in isolated cases, including tongue sensory disturbance (one case) and left-sided facial palsy (one case) (see Table 1).

**Table 1:** Detailed clinical data of 20 maxillofacial and cervical schwannomas

Case	Site	Preoperative Signs & Symptoms	Onset of Symptoms	Size (cm)	Presumptive Diagnosis	Postoperative Symptoms
1	Tongue	Foreign body sensation	7 years	1.5	Fibroma	None
2	Palate	Foreign body sensation	10 days	2.5	Papilloma	None
3	Lip	Foreign body sensation with mild tenderness	2 years	1.5	Dermoid/epidermoid cyst	Mild swelling
4	Neck	Painless, gradually enlarging mass	1 year	2.0	Schwannoma	Mild neck stiffness
5	Tongue	Painless, gradually enlarging mass	4 years	2.0	Papilloma	None
6	Neck	Pain	1 week	3.0	Schwannoma	Mild neck discomfort
7	Tongue	Hypoesthesia	8 years	1.5	Fibroma	None
8	Floor of mouth	Foreign body sensation	1 week	2.0	Sublingual gland cyst	None
9	Mandible	Swelling	8 years	10.0	Ameloblastoma	Swelling
10	Tongue	Painless, gradually enlarging mass	2 years	1.0	Fibroma	None
11	Tongue	Painless, gradually enlarging mass	1 year	2.0	Fibroma	None
12	Tongue	Painless, gradually enlarging mass	7 years	1.5	Fibroma	None
13	Lip	Painless, gradually enlarging mass	6 months	1.0	Inflammatory granuloma	Mild tenderness
14	Chin	Painless, gradually enlarging mass	6 years	2.0	Fibroma	None
15	Parotid gland	Gradually enlarging mass with pain	2 years	5.0	Pleomorphic adenoma	Mild facial swelling
16	Lip	Foreign body sensation	3 months	1.0	Inflammatory granuloma	None
17	Parotid gland	Painless, gradually enlarging mass	2 years	3.0	Pleomorphic adenoma	None
18	Neck	Painless, gradually enlarging mass	4 months	5.0	Branchial cleft cyst	Mild neck stiffness
19	Parotid gland	Left-sided facial paralysis	3 months	5.0	Adenoid cystic carcinoma	Mild facial weakness
20	Retromolar pad	Foreign body sensation affecting eating	1 year	2.5	Inflammatory granuloma	None

**Fig. 1:**
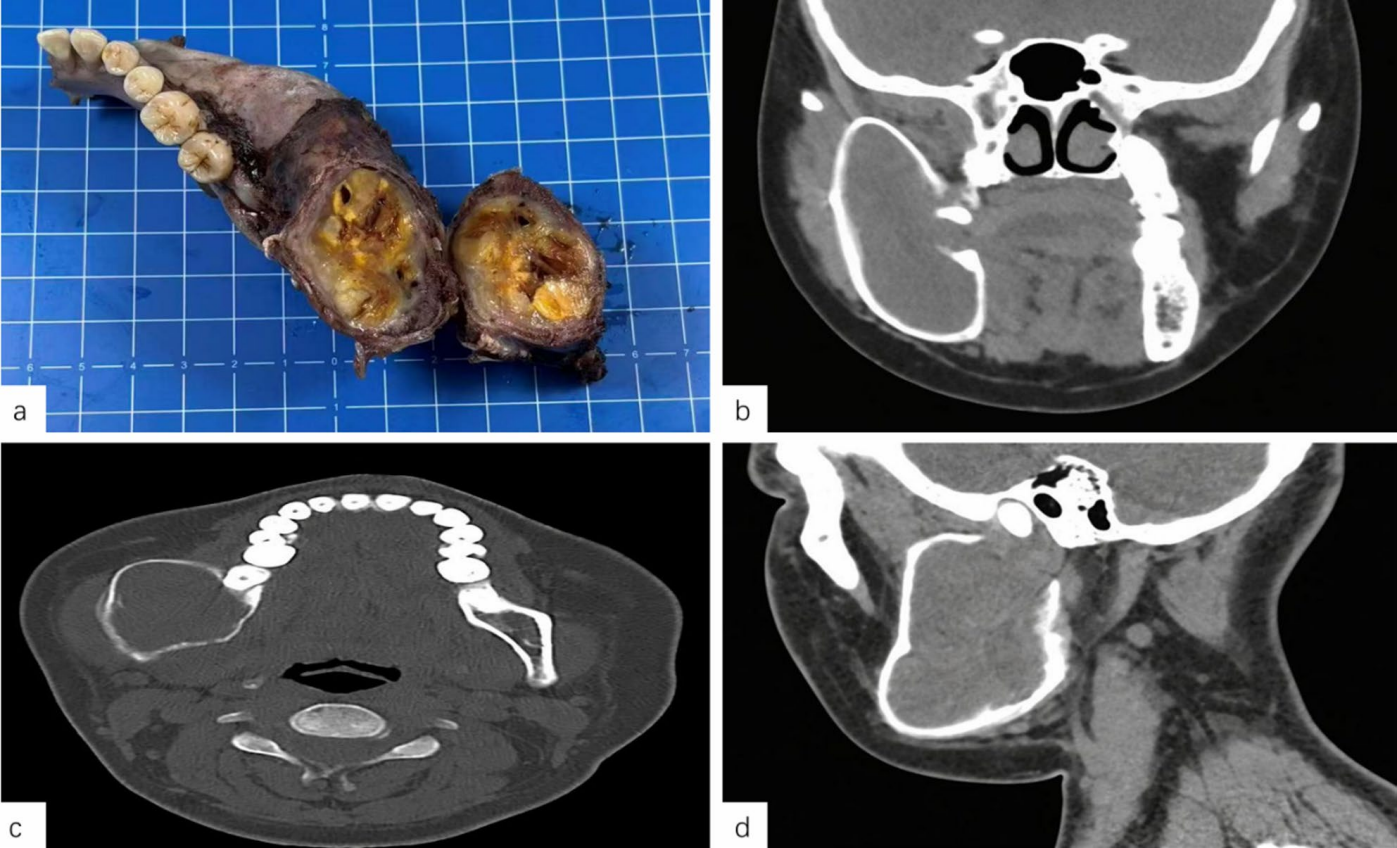
Macroscopic and radiographic features of the mandibular lesion. **a** Gross specimen of the mandibular lesion, showing a cystic-solid structure with grayish-yellow and hemorrhagic contents within the cavity. **b**–**d** Computed tomography (CT) images of the mandible reveal a well-defined, low-density mass with heterogeneous internal density, cortical expansion and thinning, focal cortical discontinuity, and adjacent bone resorption, consistent with features of an aggressive mandibular lesion

A single case involved a mandibular intraosseous SCH, presenting with notable clinical features. The patient exhibited right facial swelling with hemifacial asymmetry. On palpation, a well-defined, firm, and poorly mobile mass measuring approximately 6.0 × 10.0 cm was identified. Intraoral examination revealed marked expansion of the right mandibular body and ramus, with intact overlying mucosa and “ping-pong ball–like” consistency on palpation. The tongue mobility was preserved. The mandibular lesion and corresponding imaging features are shown in Fig. 1.

### Nerve of origin and surgical approach

**Fig. 2:**
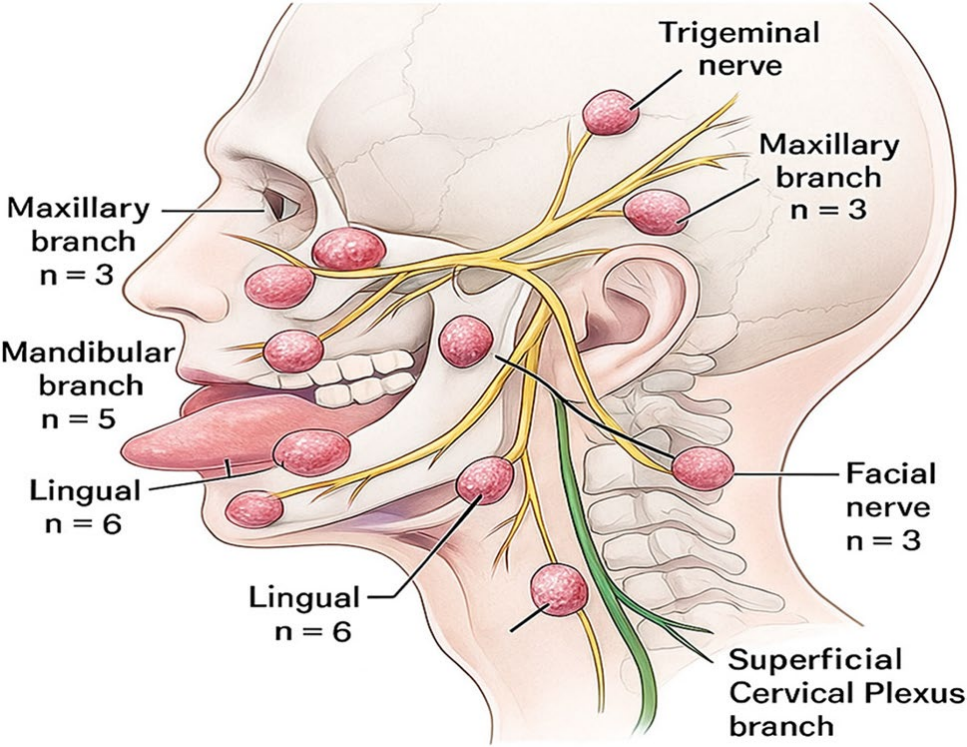
Schematic diagram showing the nerve distribution of 20 schwannoma cases: maxillary branch of the trigeminal nerve (*n* = 3), mandibular branch of the trigeminal nerve (*n* = 5), lingual nerve (*n* = 6), facial nerve (*n* = 3), and superficial branches of the cervical plexus (*n* = 3)

The presumed nerves of origin for the 20 SCHs were the maxillary branch of the trigeminal nerve (three cases), mandibular branch of the trigeminal nerve (five cases), lingual nerve (six cases), facial nerve (three cases), and superficial branches of the cervical plexus (three cases) based on tumor location and clinical presentation (Fig. 2). All patients underwent surgical resection, with intraoral approaches used in 13 cases, extraoral approaches in six cases, and a combined intraoral–extraoral approach in one case (Table 2). Most tumors were completely excised, with efforts made intraoperatively to preserve the native nerve structure and function, although, only one case exhibited mild invasion into surrounding tissues.

**Table 2:** Surgical procedure and approach

Case	Surgical Procedure	Surgical Approach	Preservation of Nerve of Origin
1–8	Complete excision	Intraoral/Extraoral	Yes
9	Digital surgical guide + Extended resection + Reconstruction plate + Vascularized fibular graft	Intraoral & Extraoral	Yes
10–20	Complete excision	Intraoral/Extraoral	Yes

Case 9 involved a mandibular intraosseous SCH with extensive involvement of the mandibular body and ramus. The preoperative multidisciplinary evaluation indicated high surgical complexity and risk, as well as a substantial mandibular defect. Surgical management included extended resection assisted by a digital surgical guide, mandibular reconstruction with a reconstruction plate, and repair using a vascularized fibula flap. The patient achieved favorable postoperative recovery.

### Histopathological features

**Fig. 3:**
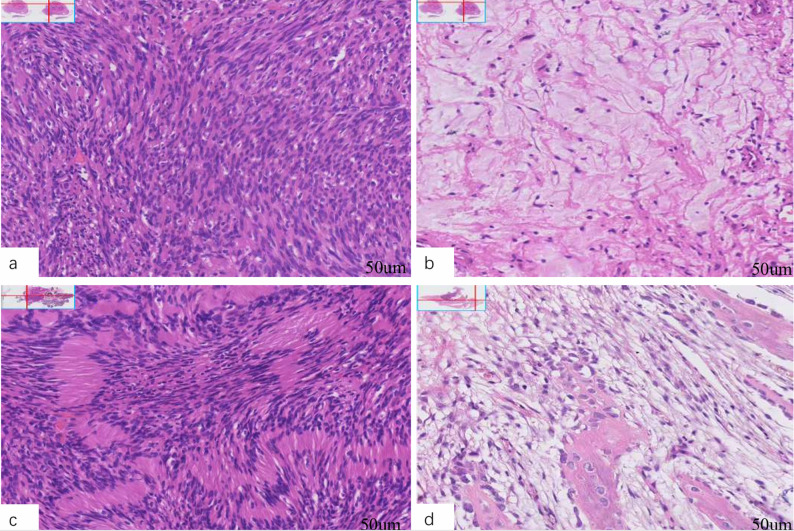
Hematoxylin and Eosin (H&E)–stained sections of oral, maxillofacial, and cervical schwannomas: **a** Antoni A area, 20×; **b** Antoni B area, 20×; **c** Verocay body, 20×; **d** tumor involvement of bone tissue, 20×

SCHs were composed of alternating hypercellular Antoni A areas and hypocellular Antoni B areas. The Antoni A regions mainly consisted of spindle-shaped cells arranged in fascicular or whorled patterns, with eosinophilic cytoplasm and elongated nuclei with blunt or rounded ends. The Antoni B regions were characterized by a loosely arranged cellular distribution within a myxoid stroma. Typical Verocay bodies were observed in four cases. All tumors were encapsulated, and the histological features were largely consistent across different anatomical sites (Fig. 3).

### Immunohistochemical findings

**Fig. 4:**
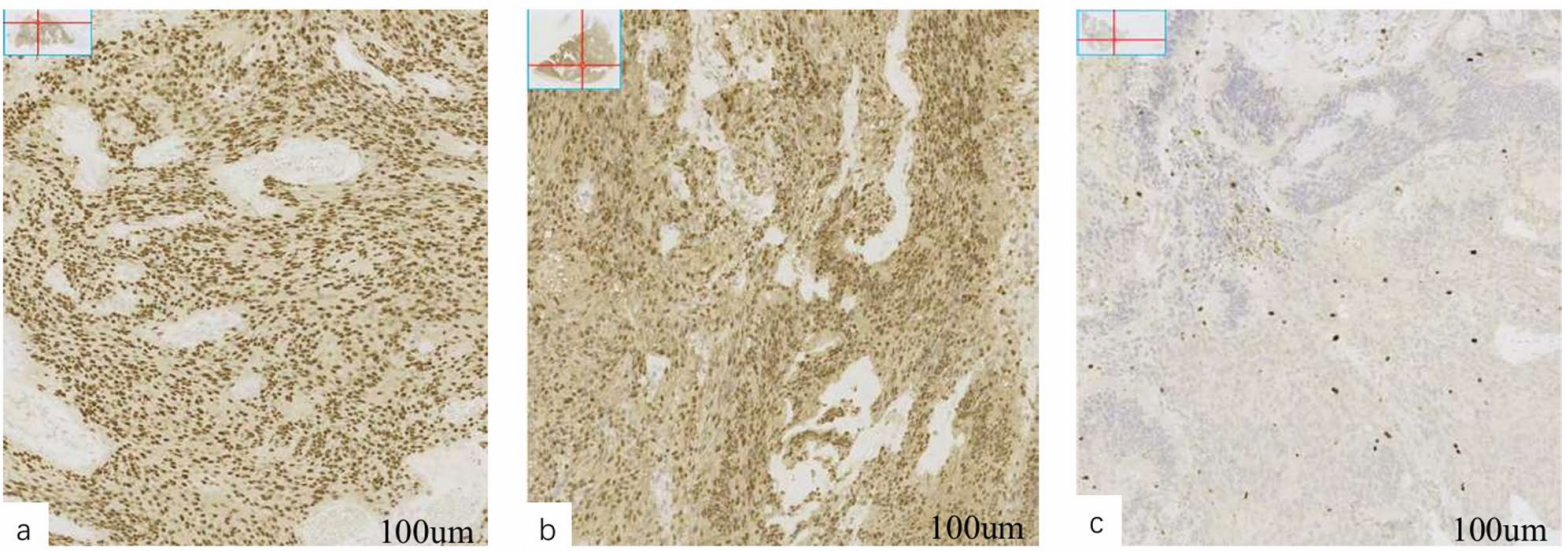
Immunohistochemical staining of oral, maxillofacial, and cervical schwannomas: **a** SOX10 showing diffuse and strong nuclear positivity, 10×; **b** S-100 showing strong cytoplasmic and nuclear positivity, 10×; **c** Ki-67 proliferation index, 10×

A total of 20 patients with oral, maxillofacial, and cervical SCHs were included in this study, of whom 11 underwent immunohistochemical analysis. The remaining cases were diagnosed based on H&E staining, pathological morphology, and clinical history. IHC demonstrated that the core SCH markers, S-100 and SOX10, were diffusely and strongly positive in all tested cases, confirming their origin from Schwann cells and serving as key diagnostic indicators for SCH. All other antibodies were negative, including AE1/AE3, Desmin, SMA, EMA, CEA, Caldesmon, MyoD1, Myogenin, Melan-A, HMB45, NSE, GFAP, CgA, and Syn. Focal positivity was observed in some cases: Vimentin (5/11, 45.5%), CD10 (4/11, 36.4%), CD34 (5/11, 45.5%), CD68 (4/11, 36.4%), CD99 (2/11, 18.2%), CK5/6 (1/11, 9.1%), STAT6 (2/11, 18.2%), Bcl-2 (7/11, 63.6%), and β-catenin (3/11, 27.3%). The Ki-67 proliferation index ranged from 2% to 5%, consistent with a benign tumor phenotype. Overall, the immunohistochemical profile was consistent with the diagnosis of SCH (Fig. 4 and Table 3).

**Table 3:** Immunohistochemical profile of 20 schwannoma cases (*n* = 11 tested)

Marker	Positive cases *n* (%)	Interpretation / Diagnostic significance
S-100	11 / 11 (100%)	Typically shows diffuse positivity, indicating Schwann cell origin, and represents the most important marker for the diagnosis of schwannoma.
SOX10	11 / 11 (100%)	A highly sensitive marker of neural crest origin; together with S-100, it constitutes the core immunophenotype of schwannoma.
AE1 / AE3	0 / 11 (0%)	Negative for epithelial markers, excluding epithelial tumors.
Vimentin	5 / 11 (45.5%)	Approximately half of schwannomas may show positivity; supportive for diagnosis but not specific.
Desmin	0 / 11 (0%)	Negative for myogenic markers, excluding rhabdomyomas or tumors of smooth muscle origin.
SMA	0 / 11 (0%)	Negative for smooth muscle markers, excluding leiomyomas.
EMA	0 / 11 (0%)	Negative for epithelial membrane antigen, excluding meningiomas and related tumors.
CEA	0 / 11 (0%)	Negative for glandular epithelial markers.
Caldesmon	0 / 11 (0%)	Excludes myogenic tumors.
MyoD1	0 / 11 (0%)	Excludes tumors of skeletal muscle origin.
Myogenin	0 / 11 (0%)	Negative for skeletal muscle differentiation.
Melan-A	0 / 11 (0%)	Negative for melanocytic markers; consistent with HMB45, excluding melanoma.
HMB45	0 / 11 (0%)	Negative for melanocytic markers.
NSE	0 / 11 (0%)	Negative for neuroendocrine markers.
GFAP	0 / 11 (0%)	Negative for oligodendrocyte and astrocyte markers.
CgA	0 / 11 (0%)	Negative for neuroendocrine markers.
Syn	0 / 11 (0%)	Negative for neuroendocrine markers.
CD10	4 / 11 (36.4%)	Focal expression observed; weak positivity seen in some soft tissue tumors.
CD34	5 / 11 (45.5%)	Approximately half of schwannomas may show positivity; supportive for diagnosis but not specific.
CD68	4 / 11 (36.4%)	Associated with histiocytic or phagocytic cells; non-specific.
CD99	2 / 11 (18.2%)	Focal weak positivity; of limited significance.
CK5/6	1 / 11 (9.1%)	Rarely weakly positive; important for excluding epithelial-origin tumors.
STAT6	2 / 11 (18.2%)	Predominantly negative; low-level positivity does not support a diagnosis of solitary fibrous tumor.
Bcl-2	7 / 11 (63.6%)	Positivity observed; however, non-specific.
β-catenin	3 / 11 (27.3%)	Predominantly membranous staining; nuclear staining is rare and non-typical.
Ki-67	2–5%	The proliferative index is low, consistent with the behavior of a benign neoplasm.

### Postoperative follow-up and prognosis

All patients in this cohort were followed for 12–24 months postoperatively. Most patients experienced favorable recovery, with no significant persistent symptoms or tumor recurrence. A small number of patients exhibited mild cervical stiffness or facial muscle weakness in the early postoperative period. Such minor neurological deficits have been reported in previous studies and generally improved gradually over time.

## Discussion

SCHs are benign peripheral nerve sheath tumors originating from Schwann cells. Schwann cells, key to peripheral nerves, form the myelin sheath for neural signal conduction and also serve as the cellular origin of peripheral, sensory, and motor nerve sheath tumors [5]. SCHs most commonly occur in the head and neck region, accounting for approximately 25–45% of cases, with the remainder distributed across other parts of the body [6]. The common sites include the flexor surfaces of the upper and lower limbs, whereas less frequent locations include the stomach and abdominal wall [7–10]. SCHs can arise from multiple tissue structures, affecting both soft and bony tissues within the oral cavity. The tongue is the most frequently involved site, followed by the palate, floor of the mouth, lips, and parotid region [11]. In the present study, tumors were observed in the tongue, upper and lower lips, chin, parotid region, neck, and mandible.

The head and neck SCHs often originate from the facial nerve and may involve any segment along its course from the brainstem to the temporal bone [12]. Facial nerve SCHs can present with diverse clinical manifestations, including tinnitus, otalgia, hearing loss, vertigo, headache, peripheral facial palsy, and trigeminal neuralgia [13]. In the present cohort, one patient presented with unilateral facial paralysis. Given the facial nerve’s motor, sensory, and parasympathetic functions, meticulous preservation of the nerve and its function during surgery is particularly critical.

These tumors may occur across a wide age range but predominantly affect young and middle-aged adults between 30 and 60 years of age [14]. In this study, the youngest patient was 11 years old. The existing literature indicates that SCHs can present at nearly any age, highlighting the variability in age distribution.

As previously noted, oral SCHs may also arise within bone. Intraosseous mandibular SCHs is a rare lesion, with clinical and radiographic features that are often non-specific and can be easily confused with odontogenic cysts or tumours. The majority of cases occur in young to middle-aged adults, with slow, painless mandibular swelling being the most common presenting symptom. Complete surgical excision is the treatment of choice, and histopathological examination combined with S-100 immunostaining remains the diagnostic gold standard. Our cases were compared with previously reported lesions (Table 4), demonstrating consistent clinical and radiographic characteristics while providing more comprehensive information on nerve origin and immunohistochemical profiles.

**Table 4:** Reported cases of intraosseous mandibular schwannoma in the literature and comparison with the present study

Author (Year)	Age / Sex	Clinical presentation	Radiological features	Treatment	Reference
Zainab et al. (2012) [15]	15 / M	Swelling of lower jaw	Expanded buccal/lingual cortex	Surgical excision	Zainab H *et al. J Oral Maxillofac Pathol*. *2012 *[15]
Kharbouch et al. (2022) [16]	57 / M	Lip paresthesia + swelling	Well-defined radiolucent lesion on X-ray	Total excision & nerve preservation	*Mandibular nerve schwannoma 2022* [16]
Alabdulkarim (2024) [17]	40 / F	Right mandibular swelling	Unilocular radiolucency extending from mandible angle	Surgical resection + reconstruction plate	Alabdulkarim *et al. J Surg Case Rep*. *2024 *[17]
Present study	37 / F	Right mandibular swelling	Unilocular radiolucent lesion involving the mandibular angle and ramus	Digital surgical guide + Extended resection + Reconstruction plate + Vascularized fibular graft	This study

Three primary hypotheses regarding the origin of intraosseous SCHs have been proposed: (1) extrinsic SCHs eroding into bone; (2) tumors arising within the nerve canal, growing in a “dumbbell” shape and causing canal enlargement; and (3) tumors originating directly from intraosseous nerve tissue [18]. In the ninth case, the tumor involved the mandibular body and ramus, and its location along the inferior alveolar nerve supports the second hypothesis.

Most head and neck SCHs remain asymptomatic unless they reach substantial size. The patients may experience dysphagia, pain, hoarseness, cranial neuropathies, or Horner syndrome depending on tumor location, mass effect, or nerve involvement. Other possible symptoms include taste disturbances and loss of motor and/or sensory function. In this study, two patients exhibited facial alterations: one had mild unilateral facial palsy (House–Brackmann II–III), and another presented with facial swelling, both affecting facial aesthetics.

Histopathological examination remains the gold standard for SCH diagnosis. SCHs can be classified into several subtypes based on histological features, including ancient, microcystic/reticular, epithelioid, cellular, granulomatous, and melanotic variants [19, 20]. Classic SCHs display the characteristic Antoni A and Antoni B growth patterns: Antoni A areas consist of densely packed spindle cells with nuclear palisading and Verocay bodies, while Antoni B areas are hypocellular with loose, myxoid stroma [21]. In this study, soft tissue SCHs often exhibited both patterns, whereas intraosseous SCHs were predominantly Antoni B type. IHC plays an important diagnostic role; diffuse, strong positivity for S-100 and SOX10 supports a Schwann cell origin. All 11 cases in this study that underwent immunohistochemical analysis demonstrated these markers, and the low Ki-67 proliferation index was consistent with benign behavior. In a subset of cases, immunohistochemical expression of CD10, CD34, STAT6 and β-catenin was assessed, but their diagnostic value should be interpreted with caution. CD34 positivity may overlap with solitary fibrous tumours (SFTs), CD10 can aid in excluding other spindle cell neoplasms, STAT6 negativity supports the exclusion of SFT, and absence of nuclear β-catenin helps distinguish fibromatous lesions. Although these markers are not specific for SCHs, their evaluation in conjunction with diffuse S-100 and SOX10 immunoreactivity and characteristic histological features enhances diagnostic reliability. SCHs should be differentiated from neurofibroma, traumatic neuroma, and SFTs: neurofibromas are typically unencapsulated with weak or patchy S-100 expression, traumatic neuromas are associated with prior nerve injury and exhibit disorganised architecture, and SFTs display branching (‘staghorn’) vessels with nuclear STAT6 positivity. In our series, weak or focal STAT6 expression was observed in some cases, but the absence of typical SFTs morphology, together with diffuse S-100/SOX10 positivity and clinical features, supported a diagnosis of SCHs.

Although SCHs are generally benign, secondary infection may occur, particularly in lesions with mucosal compromise or areas prone to contamination. Such infection can elicit local and systemic inflammatory responses, reflected in elevated C-reactive protein (CRP), leukocyte counts, or neutrophil-to-lymphocyte ratio (NLR) [22]. Assessment of these systemic markers in the present study provided additional insight into the host response to tumour-associated inflammation. Clinically, elevated inflammatory markers may correlate with pain, swelling, or erythema, helping to distinguish complicated lesions from uncomplicated SCHs. Understanding the interplay between tumour pathology and host inflammatory response can inform preoperative management, including surgical timing and the use of perioperative antibiotics, and suggests that systemic inflammatory profiling may have utility in evaluating secondary infection and in the comprehensive assessment of head and neck tumours.

SCHs can occur sporadically or as part of genetic syndromes, most notably neurofibromatosis type 2 (NF2), which is characterized by bilateral vestibular SCHs and frequent involvement of multiple cranial and spinal nerves. The head and neck region is commonly affected in NF2, whereas oral, maxillofacial, and cervical SCHs are generally sporadic. No case in this study exhibited multiple lesions, bilaterality, or family history, supporting their classification as sporadic SCHs.

Surgical excision remains the treatment of choice for oral SCHs. Intraoral approaches are preferred to avoid external scarring. However, larger tumors or those in anatomically challenging locations may require extraoral approaches. Overall, prognosis after SCH resection is excellent, and recurrence is rare if complete excision is achieved. However, long-standing, progressively enlarging lesions can significantly increase surgical complexity.

This retrospective study summarizes cases of SCHs arising in the oral, maxillofacial, and cervical regions. Given their nonspecific clinical presentation and relative rarity in the head and neck, diagnosis can be challenging and ultimately relies on histopathology and IHC. Postoperative follow-up is recommended to monitor for recurrence. Through the combination of case analysis with a systematic literature review, this study provides insights into the diagnostic features, surgical management, and histopathological characteristics of oral and maxillofacial SCHs, offering practical guidance for clinical practice.

## Supplementary Information

Supplementary Material 1.

## Data Availability

The datasets generated and/or analyzed during the current study are available from the corresponding author on reasonable request.

## Abbreviations

SCH: Schwannoma
CT: Computed tomography
IHC: Immunohistochemistry
