# Supplementary material for: Oral–maxillofacial and cervical schwannomas: a retrospective cohort analysis with a rare intraosseous case
Source: BMC Oral Health. 2026 Mar 9;26:676. doi: 10.1186/s12903-026-08073-4 (PMC13085569; doi:10.1186/s12903-026-08073-4)
Supplement: Supplementary file 1 — Supplementary Material 1. [file 12903_2026_8073_MOESM1_ESM.pdf]

贵州医科大学附属口腔医院医学伦理委员会

伦理审查批件

The Ethics Committee of GuiZhou Medical University

Ethics Approval Document

批件号（Approval Number）：2025 伦审第（ 46 ）号

**项目名称**<br>**(Protocol Name)**	头颈部神经鞘瘤临床与病理特征回顾性研究<br>Retrospective Study of Clinical, Pathological, and Immunohistochemical Features of Head and Neck Schwannomas		
**项目类别**<br>**(Protocol Source)**	□A.基础研究（basic research）<br>□B.应用基础研究（basic research for application)<br>□C.临床研究(clinical research)<br>□D.药学研究（pharmacy research)<br>□E.新技术应用(New technology test)<br>□F.限制类技术（Restricted technology)<br>□G.其他(Other)（请注明 please specify）		
**申请部门**<br>**(Research Department)**	病理科<br>Department of Pathology	**部门负责人**<br>**(Deputy of Department)**	吴亚东<br>WU YA DONG
**项目负责人**<br>**(Principle Investigator)**	胡小敏<br>HU XIAO MIN		
**研究目的**<br>**(Aim of Project)**	评估口颌面及颈部神经鞘瘤的临床、病理及免疫组化特征，为诊断与管理提供参考。To evaluate the clinical, pathological, and immunohistochemical features of head and neck schwannomas to inform diagnosis and management.		
**涉及人体研究内容**<br>**(The Contents Related to *in vivo* Human Study)**	本研究回顾性纳入 20 例头颈部神经鞘瘤患者（男性 7 例，女性 13 例）。纳入标准：①肿瘤位于口腔颌面及颈部；②组织病理学确诊；③石蜡包埋标本可用于免疫组织化学；④临床资料完整。排除标准：①非头颈部肿瘤；②病理标本或资料不全；③随访信息缺失。方法：系统收集病史、临床表现及辅助检查资料，行 HE 及免疫组化染色明确诊断，并对人口学特征、临床表现、手术方式及病理结果进行描述性分析。This retrospective study included 20 patients with head and neck schwannomas (7 males and 13 females). Inclusion criteria were: (1) tumor located in the oral–maxillofacial region or neck; (2) histopathologically confirmed diagnosis; (3) paraffin-embedded tissue suitable for immunohistochemistry; and (4) complete clinical data. Exclusion criteria were: (1) tumor outside the head and neck region; (2) incomplete pathological specimens or data; and (3) missing follow-up information. Clinical history, presentation, and ancillary examination data were systematically collected. Hematoxylin–eosin and immunohistochemical staining were performed to confirm the diagnosis, and descriptive analyses were conducted on demographic characteristics, clinical features, surgical approaches, and pathological findings.		
**可能出现的不良反应与危害和防治与补偿措施**<br>(Possible Adverse Reactions and Hazards and Prevention and Compensation Measures)	该研究是回顾性的研究，没有使用干预性的实验方案。受试者权益得到充分保护，该项目不增加受试者的医疗费用和痛苦，对受试者不存在潜在风险。<br>The study was retrospective and did not use interventional protocols. Subjects 'rights are fully protected. The project does not increase subjects' medical costs and pain, and there is no potential risk to subjects.		

CS 扫描全能王
3亿人都在用的扫描App

申请人（项目负责人）承诺：

以上所填内容属实，本人承诺待该项目批准后，我将遵循 GCP、方案以及伦理委员会的要求，开展本项临床研究。所有涉及人类遗传资源采集、收集、买卖、出口、出境的研究，待获得人类遗传办批件后再开展。

The Promises of Applicant or Principle Investigator:

The above contents are true. If approved, I will strictly abide by the "Articles of Association of the Human Ethics Committee of Guizhou Medical University" and conduct project research according to the provided scheme.

签字(Sign)：胡小敏　　日期(Date)：2025.12.10.

审查途径<br>**Approval Category**	快速审查(Quick Review)☐✓　　书面审查(Written Review) ☐
	会议审查(Meeting Review)☐　　会议日期(Meeting Date)：
	结论(Result)：<br>✓☐同意 (approval)　　☐修改后同意(approval after revising)<br>☐不同意 (disapproval)　　☐修改后重审(Appraisal after revising)<br>☐暂停已批准的试验(Terminateor suspend its prior approval)

审查意见(Review Recommendation)：

经我校伦理委员会讨论审核，认为该项目符合《涉及人的生物医学研究伦理审查办法（试行）》及赫尔辛基宣言关于生物人体试验的相关规定，同意在我校收集样本开展本项研究。

After discussion or review by the ethics committee of our hospital, the project was found to be in compliance with the Ministry of Health's "《Methods for Ethical Review of Biomedical Research Involving Human Beings (Trial)》" and the relevant provisions of the Helsinki Declaration on biological human testing, and agreed to collect samples from our hospital for this research.

主任委员签字：

(Signature by the Ethics Chairman)

盖章 (Stamp)

日期(Date) 2025.12.10

附：申请人（项目负责人）简要信息

Attachment: Information of Applicant or Principle Investigator

姓名<br>(Name)	胡小敏<br>HU XIAO MIN	性别<br>Sex	女<br>Female	学历学位<br>Education Background	硕士<br>Master	职称<br>Title	住院医师<br>Resident physician
电话(Phone)	0851-88549472			E-mail		3436223926@qq.com	
手机(Mobile Phone)	15885776322			邮编(Zip Code)		550000	
通讯地址<br>Correspondence Address	贵州省贵阳市云岩区北京路 9 号<br>No. 9 Beijing Road, Yunyan District, Guiyang, Guizhou Province, China						
研究方向<br>Research Interest	口腔颌面骨和软骨组织再生<br>Regeneration of oral and maxillofacial bone and cartilage tissues						

声明：本伦理委员会的组成、职责及工作程序遵循中国 GCP 和有关法规。

地址(Add.)：贵州省贵阳市云岩区北京路 9 号贵州医科大学附属口腔医院

电话(Tel.)：+86 0851-88416075

本申请表一式两份，一份交项目负责人保管，一份交科研管理科留存归档

CS 扫描全能王
3亿人都在用的扫描App
